# Persistent neurocognitive deficits in cognitively impaired survivors of sepsis are explained by reductions in working memory capacity

**DOI:** 10.3389/fpsyg.2024.1321145

**Published:** 2024-02-21

**Authors:** Fabian Kattlun, Elizabeth Hertel, Christian Geis, André Scherag, Jonathan Wickel, Kathrin Finke

**Affiliations:** ^1^Department of Neurology, Jena University Hospital - Friedrich Schiller University, Jena, Germany; ^2^Center for Sepsis Control and Care, Jena University Hospital - Friedrich Schiller University, Jena, Germany; ^3^Section Translational Neuroimmunology, Department of Neurology, Jena University Hospital - Friedrich Schiller University, Jena, Germany; ^4^Institute of Medical Statistics, Computer and Data Sciences, Jena University Hospital - Friedrich Schiller University, Jena, Germany

**Keywords:** sepsis, cognition, neuropsychological impairment, visual attention, working memory

## Abstract

**Introduction:**

Sepsis is defined as life-threatening organ dysfunction caused by a dysregulated host response to infection. Mounting evidence suggests that many cognitively impaired sepsis survivors show long-term neurocognitive deficits in neuropsychological tasks. To date, the underlying mechanisms of these deficits are insufficiently understood. Based on previous evaluations we hypothesized that visual attention and working memory may be affected in a sample of cognitively impaired sepsis survivors.

**Methods:**

We utilized psychophysical whole-and partial-report paradigms based on the computational theory of visual attention (TVA) to determine (i) whether sepsis survivors show changes in basic parameters of visual attention and working memory, (ii) whether the affected parameters are related to neuropsychological test results in a standard battery in sepsis survivors and matched healthy control participants, (iii) whether between-group differences in these basic parameters of visual attention could account for underperformance of sepsis survivors in neuropsychological tests when adjusting for potentially relevant clinical variables.

**Results:**

We showed that, in sepsis survivors, the maximum number of elements consciously maintained in an instant, i.e. the working memory storage capacity *K*, is reduced (sepsis survivors: *M* = 3.0; healthy controls: *M* = 3.4). Moreover, *K* explained variance in neurocognitive outcomes –17% in attentional and 16 % in executive functions – in a standard neuropsychological battery. The association remained stable when adjusting for clinical variables.

**Discussion:**

Thus, in our sample of cognitively impaired sepsis survivors, a reduction in working memory capacity seems to be a critical determinant of the neurocognitive sequelae. It should be the subject of future work on mechanisms but may also serve as surrogate outcome measure in interventional studies.

## 1 Introduction

Sepsis is a life-threatening organ dysfunction evoked by a severe immune response to an infection (Singer et al., 2016). In 2017, around 49 million incident cases of sepsis were recorded worldwide (Rudd et al., 2020). During the acute stage, most patients exhibit sepsis-associated encephalopathy with clinical manifestations ranging from mild delirium to coma (Atterton et al., 2020). Moreover, a substantial number of sepsis survivors develop chronic and permanent sequelae, e.g., 24–36% show long-term neurocognitive deficits 1 year after discharge (Pandharipande et al., 2013). As shown in a longitudinal population-based cohort study such deficits are also found in patients without prior impairment (Iwashyna et al., 2010). Thus, sepsis seems to causally induce cognitive impairment. In the Mid-German Sepsis Cohort (MSC) (Scherag et al., 2017; Fleischmann-Struzek et al., 2021) assessed here, which was established following the acute phase, such a causal relationship cannot be drawn. It is important to note, however, that the incidence of cognitive impairment in the MSC of 21.3% (Stallmach et al., 2022) is equivalent to Iwashyna et al. (2010) and also to other studies (Davydow et al., 2012; Iwashyna et al., 2012).

Identified risk factors for the occurrence of post-sepsis neurocognitive dysfunction are the duration of delirium (Pandharipande et al., 2013), depressive symptoms and the lengths of hospitalization (Calsavara et al., 2018). Cognitive deficits are clinically highly relevant, as they lead to significant functional and psychosocial decline of the affected individuals, which also results in substantial burden for their primary caregivers. In particular, they lead to a reduced probability of being able to return to work, to participate in social and family activities, and to experience an overall diminished health-related quality of life (Iwashyna et al., 2010; Lazosky et al., 2010; Prescott and Angus, 2018; König et al., 2019; Prescott et al., 2019; Stallmach et al., 2022).

So far, the nature of the cognitive deficits in sepsis survivors is not well understood. Deficits have been reported primarily in tasks targeting visual perception, attention, executive and short-term memory functions (for review see Calsavara et al., 2018). Impairments in these domains are broadly in line with a magnetoencephalography study showing that lasting cognitive deficits are related to abnormal dynamics within the thalamo-cortical brain network (Götz et al., 2016), i.e., a network with known relevance for visual attentional and short-term memory processes (Bundesen et al., 2005; Menegaux et al., 2020). However, successful performance in established clinical neuropsychological tasks typically relies on the integrity of diverse basic functions, such as, e.g., processing speed, working memory and top-down control of selection. Thus, for understanding the basis of broad neuropsychological dysfunction as measured by standard tasks, such basic parameters need to be evaluated. Identification of such underlying mechanisms could result in quantifiable neurocognitive biomarkers. These might be useful for tailored neurocognitive interventions, for patient stratification in intervention studies, and for outcome evaluation of intervention efficacy.

A more systematic assessment of the underlying basic mechanisms can be achieved by the computational theory of visual attention (TVA; Bundesen, 1990). In TVA, visual processing is conceived as a race of visual objects toward selection, that is, representation in a capacity-limited visual working memory store. The race is determined by the speed of processing of the individual objects. It is terminated when the working memory store is filled up to its limits. Only objects that reached the store are consciously available for further actions, such as verbal report. According to the neural interpretation of TVA (the neural TVA, NTVA), these visual attention and particularly working memory functions rely on the integrity of visual thalamic and occipital, temporal and parietal cortical structures, and their connectivity. Critically, based on TVA, distinct parameters of visual attention and working memory that determine a given participant’s visual attentional performance can be estimated based on performance in two psychophysical tasks. In a whole report task, with visual arrays containing multiple letter stimuli are briefly presented on a computer screen. The participants are instructed to report as many as letters as possible. In a partial report task, subjects have to report pre-specified (i.e., with respect to color) target letters only while ignoring distractors (see Methods for more details). Fitting the accuracy of the verbal report across the different experimental conditions in both paradigms delivers estimates of mathematically independent and process-pure parameters of attentional and working memory capacity (whole report) and selectivity (partial report). More specifically, the resulting parameter estimates are visual threshold *t0* in ms, visual processing speed *C* in elements/s, visual working memory storage capacity *K* in number of elements (whole report) and top-down control of selection *α* (partial report). In the assessment of cognitive deficits of patients, the TVA-based method offers critical advantages reviewed, e.g., by Habekost (2015). Its excellent reliability has been demonstrated by showing low measurement errors according to bootstrapping analyses in the assessment of healthy individual and patients and highly robust parameter fits resulting even from shorter versions of the tasks that are applicable also to patients (Habekost and Bundesen, 2003; Finke et al., 2005; Habekost and Rostrup, 2006). Second, the different parameters are cognitively specific. This has been demonstrated, e.g., by selective impairments, such as in top-down control following frontal brain damage (Bublak et al., 2005) or in working memory capacity in adults with attention-deficit-hyperactivity disorder (Finke et al., 2010), by selective enhancement of processing speed by alertness cues (Haupt et al., 2018) and by alertness training (Penning et al., 2021) and finally by distinct correspondences of the different parameters to different brain correlates (Wiegand et al., 2014; Ruiz-Rizzo et al., 2018). Based on the adjustment of individual exposure durations the paradigm can be applied to different populations with diverse attentional capabilities. Thus, the TVA-based assessment is on the one hand sensitive enough to differentiate validly between relatively low and high healthy young performers as evidenced by significant correlations to established measures of attentional performance (Finke et al., 2005) and can also demonstrate subtle attentional deficits in patients that are otherwise undetected in established clinical tasks (Habekost and Bundesen, 2003). On the other hand, it can also be used to unravel the underlying basic attentional decline responsible for severe performance deficits in highly impaired and elderly patients. This was shown, e.g., in patients suffering a decline in the perception of complex visual information due to posterior cortical atrophy. This study revealed slowing of visual processing speed as the decisive underlying mechanism of the performance deficits (Neitzel et al., 2016). Performance in clinical neuropsychological tasks often relies on diverse attentional and working memory functions without distinguishing their contributions. In contrast, the TVA-based approach enables the identification of specific impaired attentional functions based on performance in a single task, thereby elucidating the basic mechanisms behind observable cognitive failures. Finally, its foundation in state-of-the-art theory also lends it high validity.

Here we investigated (i) whether survivors of sepsis with cognitive impairments suffer from changes in basic parameters of visual attention and working memory, (ii) whether the affected parameters are related to neuropsychological test results in a standard battery in survivors of sepsis and healthy control participants, thereby demonstrating the relevance of these basic parameters, and (iii) whether between-group differences in these parameters could account for the cognitive deficits of sepsis survivors, when taking account for potentially relevant clinical variables, such as depression and anxiety (Desai et al., 2011; Huang et al., 2019; Calsavara et al., 2021).

## 2 Methods

### 2.1 Participants

**Figure 1:**
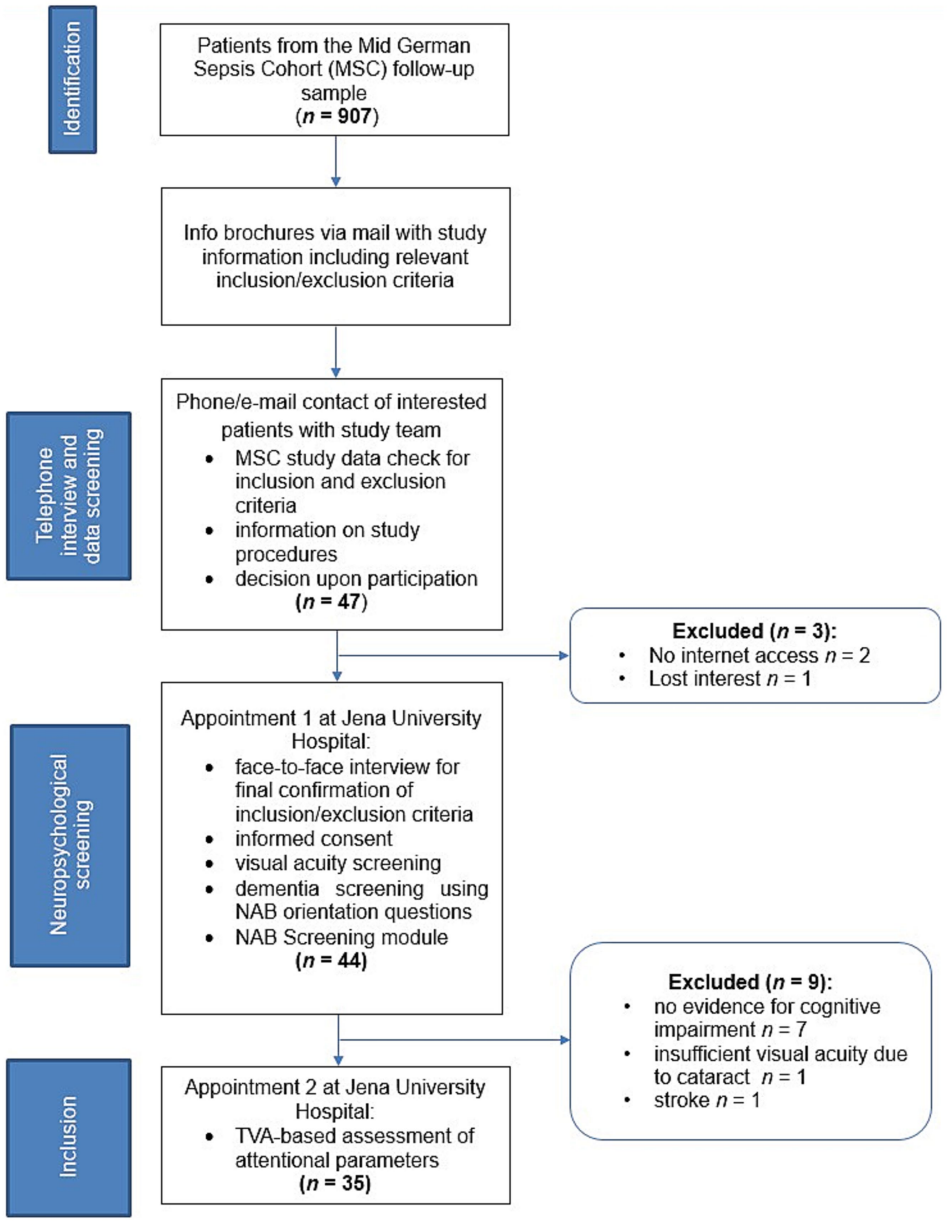
Flow-chart of the study recruitment process. MLCST, MARS Letter Contrast Sensitivity Test; NAB, Neuropsychological Assessment Battery; TVA, Theory of Visual Attention.

The recruitment procedure is shown in Figure 1. Patients were recruited from late 2019 to late 2020 from the MSC, a multicenter patient cohort on sepsis survivorship (Scherag et al., 2017; Fleischmann-Struzek et al., 2021). The MSC includes adult (i.e., aged ≥18 years) patients who were treated for sepsis or septic shock in intensive care units (ICUs). MSC research aims at quantifying mid-term and long-term functional disabilities after ICU-treated sepsis. Of the 3,210 patients initially enrolled, 907 survivors of sepsis participated in follow-up assessments.

Inclusion criteria for participation in STARDUsT^1^ were being a member of the MSC, age of 18–80 years, normal or corrected-to-normal vision and an internet connection at home. Exclusion criteria comprised patients suffering from pre-sepsis and post-sepsis dementia, severe neurological and/or psychiatric disorders or non-corrected visual impairment.

Basic demographic and clinical data available from the MSC included major neurological/psychiatric disorders such as information on pre- or post-sepsis dementia (Fleischmann-Struzek et al., 2021). Patients were screened for post-sepsis dementia during regular follow-up interviews. This included patient’s cognitive evaluations by relatives and proxies using the Informant Questionnaire on Cognitive Decline in the Elderly (IQCODE) to obtain potential new diagnoses of dementia (Scherag et al., 2017). Furthermore, interviewers qualitatively evaluated the patients’ answers in the Telephone Montreal-Cognitive Assessment (tMoCA; Katz et al., 2021) for potential signs of dementia in the orientation questions.

The STARDUsT study was announced to the MSC via newsletters that are regularly sent to the patients. Therein, MSC member were informed about the inclusion and exclusion criteria and received the basic study information. Forty-seven patients contacted the study team via telephone or e-mail. Their MSC study data were checked for inclusion and exclusion criteria and they were informed about the study procedures and then decided upon participation. Two patients did not have internet access and were excluded from participation. One patient decided not to participate following verbal description of the study procedure.

Upon agreement to participate, we scheduled an appointment at the Jena University Hospital Memory Center. During this face-to-face assessment we first re-confirmed all in- and exclusion criteria in a verbal interview. One patient reported suffering from stroke and was thus excluded.^2^ All patients gave written informed consent for study participation. Then, spatial and temporal orientation questions of the screening module of the Neuropsychological Assessment Battery (NAB; Petermann et al., 2016) were applied to confirm that basic orientation was preserved. All patients reached scores ≥26/28 and, thus, we did not have to assume overlooked manifest dementia in the invited patients. Furthermore, a visual acuity screening was used to confirm preserved basic visual perception. One patient suffered from visual loss due to cataract and was excluded.

Then, the NAB screening module was applied. Patients were excluded if they scored normal in all different cognitive domains according to the subscores of the neuropsychological test battery (*T*-value > 40). This was done as the study aim was to characterize the underlying basic mechanisms of neuro-cognitive sequelae and, thus, we were particularly interested in patients who suffer from persistent impairments following sepsis. Seven patients showed no evidence for neurocognitive impairments and were thus not included. Therefore, the final sample consisted of 35 participants or 83% of the eligible sample (age: 57.0 ± 13.9; 14 females; 10.4 ± 1.0 years of education).

An age- and education-matched healthy control group (*n* = 38) was recruited from early 2020 to late 2021. We recruited participants via phone from existing healthy volunteer participant panels of whom we had relevant sociodemographic information. This volunteer panel was established via the Jena University Hospital memory center where patients’ relatives are regularly asked whether they would be willing to and interested in participating in research studies. Additionally, in order to include a comparable number of healthy participants without an academic background we made paper announcements in public places. All control participants were naïve with respect to the specific assessment applied in the STARDUst study. Three control participants had to be excluded due to medical reasons (i.e., psychiatric disorders: *n* = 2; vision impairment: *n* = 1). Thus, the control sample consisted of 35 participants (age: 53.6 ± 12.0; 18 females; 10.5 ± 0.9 years of education). Due to technical issues, one participant in the sepsis group could not complete the TVA-based whole-report task. Table 1 summarizes the demographic and clinical characteristics of both groups. The sample assessed here was similar to the original sample of 907 survivors with respect to sex distribution, ICU stay duration and duration of ventilation. However, they tended to be younger and have lower duration of delirium. These differences are most probably due to the fact that part especially of the older MSC patients had died in the meanwhile or where not mobile enough to participate in the study (sociodemographic and clinical details of the current sample and the MSC group are listed in Supplementary Table S2). All participants gave written informed consent before taking part in the study and received monetary compensation for their participation. The study was approved by the ethics committee of the Jena University Hospital (Reg.-Nr. 2019-1411_1-BO).

**Table 1:** Demographic and medical data.

Variable	Sepsis survivors (*n* = 35)	Healthy controls (*n* = 35)	*T*-value*	*p*-value**
Age	57.0, 13.9 (33–79)	53.6, 12.0 (32–78)	−1.11	0.273
Sex (female/male)	14/21	18/17	1.91	0.168
Education in years	10.4, 1.0 (8–12)	10.5, 0.9 (9–12)	0.49	0.627
MWT-B-IQ	94.3, 10.8 (77–126)	98.2, 10.4 (83–119)	1.52	0.132
MLCST	1.72, 0.1 (1.32–1.80)	1.74, 0.1 (1.40–1.92)	1.12	0.267
HADS-D depression	6.5, 3.4 (1–15)	3.7, 3.4 (0–14)	−2.81	0.001
HADS-D anxiety	7.2, 3.9 (0–16)	3.9, 3.9 (0–14)	−3.24	0.001
ICU treatment days	16.0, 14.6 (1–64)	–	–	–
Time interval since ICU discharge in months	28.6, 12.3 (9–48)	–	–	–
Ventilation days	9.5, 11.8 (0–40)	–	–	–
Days of delirium	2.0, 3.8 (0–18)	–	–	–

Mean, SD, and range in brackets.*Chi square statistics for sex and t-statistics otherwise.**Two-sided *p*-values from the Chi square test for sex and from independent *t*-tests otherwise.HADS-D, Hospital Anxiety and Depression Scale – German Version; ICU, Intensive Care Unit; MLCST, MARS Letter Contrast Sensitivity Test; MWT-B, “Mehrfachwahl-Wortschatz-Intelligenztest.” Scores adjusted as proposed in Satzger et al. (2002).

### 2.2 General procedure

Upon arrival at the Jena University Hospital Memory Center, a trained psychologist assessed contrast sensitivity (MARS Letter Contrast Sensitivity Test, MLCST; MARS Perceptrix Corporation, Chappaqua, NY) and vocabulary knowledge as an estimate of premorbid verbal intelligence (Mehrfachwahl-Wortschatz-Intelligenztest, MWT-B; Lehrl et al., 1995), administered the screening module of the NAB (Petermann et al., 2016) and the Hospital Anxiety and Depression Scale – German Version, HADS-D (Herrmann-Lingen et al., 2011). In a separate session on another day, the TVA-based whole- and partial-report experiments were applied. Both sessions usually took place within 1 week. Each of the two sessions lasted approximately 90 min.

### 2.3 Instruments

#### 2.3.1 MLCST

The MLCST was used to assess participants’ visual peak contrast sensitivity based on a printed chart with eight rows of letters with gradually fading contrast that have to be read out.

#### 2.3.2 MWT-B

The MWT-B is a multiple-choice German vocabulary test which was used to estimate premorbid verbal intelligence. Derived IQ values have been shown to overestimate verbal intelligence, so our data have been adjusted as proposed by Satzger et al. (2002).^3^

#### 2.3.3 NAB

The screening module of the German version of the NAB consists of 14 normed subtests for the 5 cognitive domains of attention (subtests: digit span forwards, digit span backwards, digits & letters A—discriminating letter A in a digit/letter array, digits & letters B—discriminating letter A in a digit/letter array while summing up digits), language (subtests: denominating pictures, speech production), memory (subtests: shapes—immediate recall, shapes—delayed recall, story—immediate recall, story—delayed recall), perception (subtests: discriminating pictures, replicating geometric shapes), and executive functions (subtests: mazes, word fluency “P-words”). For all tests, standard values (*M* = 100, *SD* = 15) were calculated according to the manual.

#### 2.3.4 HADS-D

HADS-D was administered to assess psychological burden. Its two subscales measure anxiety and depression with seven items each. Each item has a range of zero to three, leading to subscale maximum sum scores of 15.

#### 2.3.5 TVA framework

TVA is a mathematical model (Bundesen, 1990; Bundesen et al., 2005) employing the ‘biased-competition’ framework of visual attention (Desimone and Duncan, 1995; Kastner and Ungerleider, 2000). The model views visual processing as a simultaneous and competitive race among objects. These objects compete to be chosen for awareness or conscious recognition in a visual working memory with limited capacity. Bias signals determine “attentional weights” for the objects. Depending on their relative weights, some objects are favored for selection, either automatically in a “bottom-up” manner or by an intentional, “top-down” process. Bottom-up influences stem from stimulus saliency, while top-down influences originate, e.g., from specific task instructions. The probability of selection, i.e., conscious representation, is determined by an object’s processing rate *v*, which depends on the attentional weight (*w*) assigned to it, and by the capacity of the working memory store (if the store is filled, the selection process terminates). Based on TVA, two experimental paradigms, whole- and partial-report, have been established that allow quantification of a set of attentional parameters determining visual attentional performance in a given participant.

##### 2.3.5.1 General procedure for TVA-based whole- and partial-report

Each participant completed the whole- and partial-report assessment with the first lasting approximately 60 and the latter lasting 30 min, within one testing session. In both experiments, the participants were instructed to first fixate a central white point (0.9 × 0.9 cm) presented for 1,000 ms in the partial report task and 600 ms in the whole-report task. Then, red and/or blue letters, each 1.2 cm high and 1.0 cm wide, were briefly shown against a black background. The exposure time for each letter was set during a practice session to reach a specific criterion (details below). Each trial featured randomly selected letters from a set of 23 (ABCDEFGHJKLMNOPRSTUVWXZ), with no repeats in a single trial. The letters were displayed either with or without a mask. In unmasked conditions, ‘iconic’ memory allows the letters to be seen for a few hundred milliseconds longer (Sperling, 1960). Pattern masks are thought to disrupt this memory effect. Participants could report the letters in any order, at their own pace. The experimenter recorded responses and initiated the next trial. For more details, see Supplementary material 1.1.

##### 2.3.5.2 TVA-based whole-report paradigm

**Figure 2:**
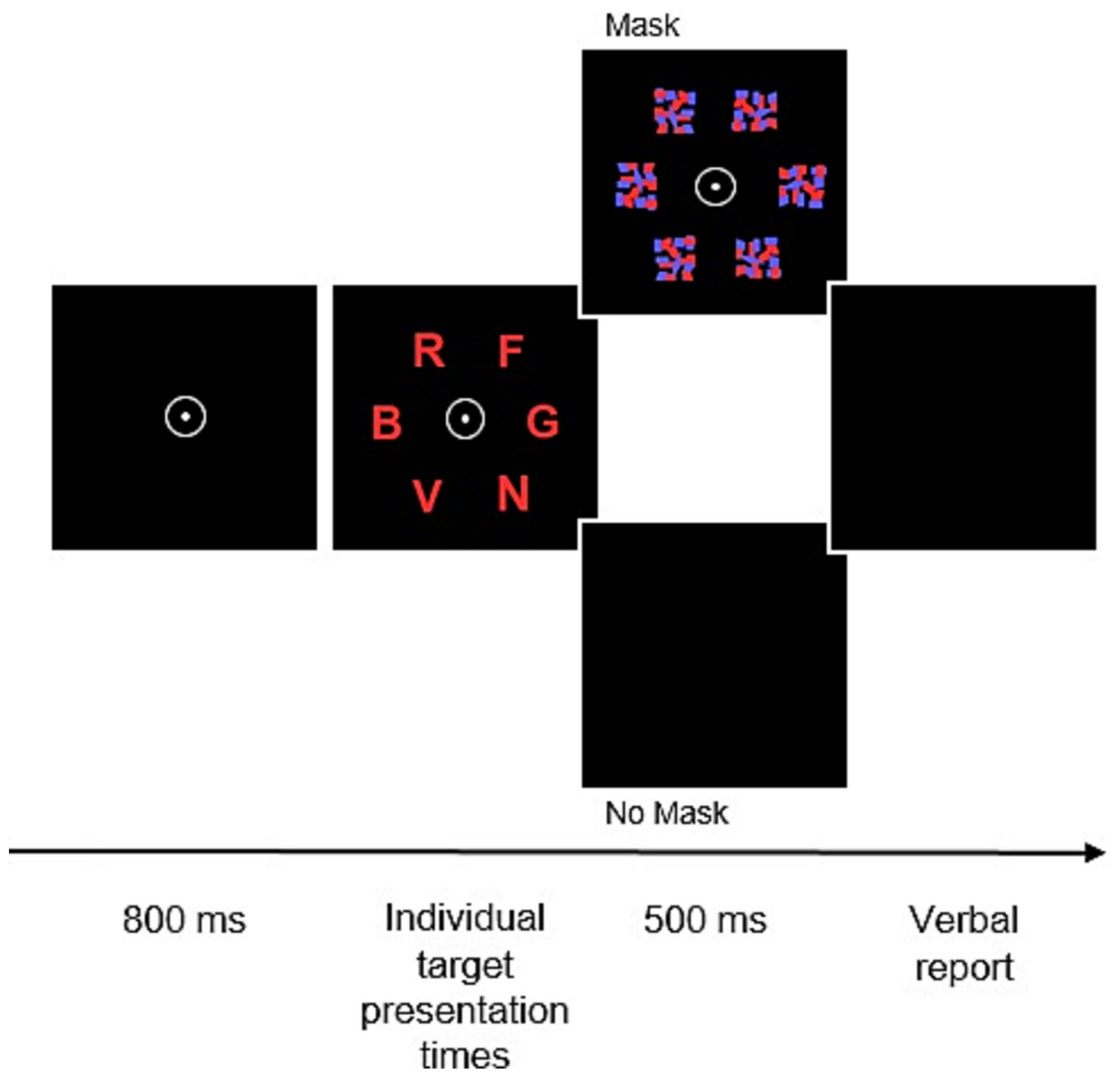
Trial sequence in the TVA-based whole-report task. After the presentation of a central fixation point for 800 ms, six random letters from a prespecified set are simultaneously flashed for predetermined individual presentation times. Following that, presented stimuli are either masked or remain unmasked for 500 ms before participants are asked to verbally report the letters.

**Figure 3:**
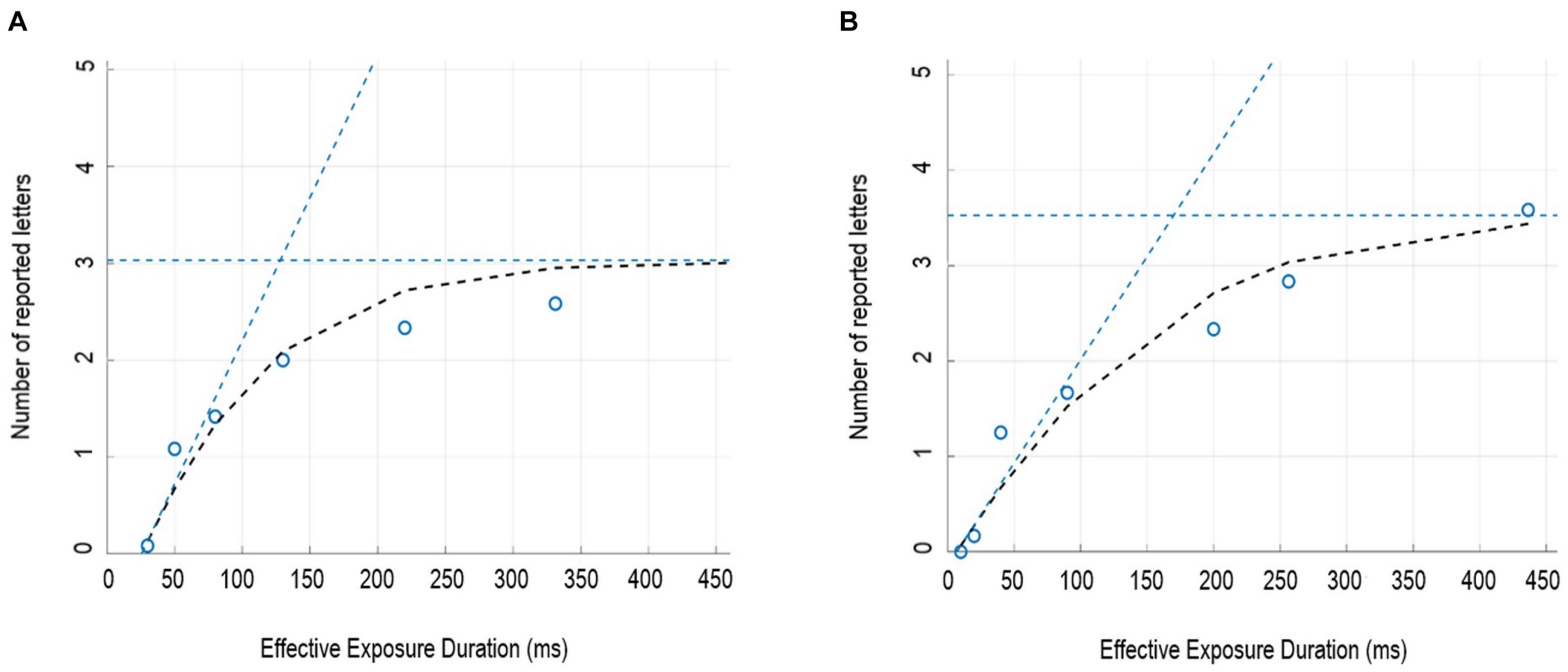
Whole-report performance for a representative sepsis survivor **(A)** and a representative, age-matched healthy control **(B)**. Depicted is the mean number of correctly reported letters as a function of effective exposure duration. Black dashed curves represent the best fits from the TVA model to the observations. Below the visual perception threshold, minimum effective exposure duration *t0*, the score is zero. At longer exposure durations the score gradually increases, following an exponential function of the presentation time. The amount of improvement with longer exposure time reflects the rate of information uptake; more precisely, the slope of the curve at x = *t0* can be taken as an estimate of the visual processing speed, *C*. The estimate of working memory storage capacity *K* is marked by the blue dashed horizontal line. The asymptote of the healthy control participant indicates a working memory storage capacity of around 3.6 elements; by comparison, the patient’s asymptote is lower, representing a lower number of elements—around 3.0—that can be represented in working memory. However, the origin of the curve (i.e., *t0*) and the slope of the curve at *t0* (i.e., *C*) are similar for both representative participants.

On each trial of the TVA-based whole-report paradigm, 6 equidistant target letters were flashed on an imaginary circle around the central fixation point (see Figure 2). Participants were advised to report all letters recognized with “fair certainty” (see Supplementary material). Five different target exposure durations were utilized. Exposure durations were determined in a pretest (see Supplementary materials 1.3, 1.4). Fitting of whole-report accuracy in the different experimental conditions yields the parameters visual threshold (minimum effective exposure duration in ms), visual processing speed (in elements/s) and working memory storage capacity (in number of elements). Detailed underlying estimation algorithms were described by Kyllingsbaek (2006). Given an object x, the probability of recognizing this specific object is mathematically modeled by an exponential growth function which is relating accuracy of report (mean number of reported elements) to effective exposure duration. The visual perceptual threshold, denoted as *t0*, is defined at the origin of the function, corresponding to the coordinates (*t0*, 0). The slope of the function at this specific point reflects the visual processing speed, represented as *C*. Additionally, the function’s asymptotic nature suggests a limit to the amount of information that can be held in working memory. The maximum quantity of items that can be represented, which defines the working memory capacity (*K*), corresponds to the level of the asymptote, as illustrated in Figure 3.

##### 2.3.5.3 TVA-based partial report paradigm

**Figure 4:**
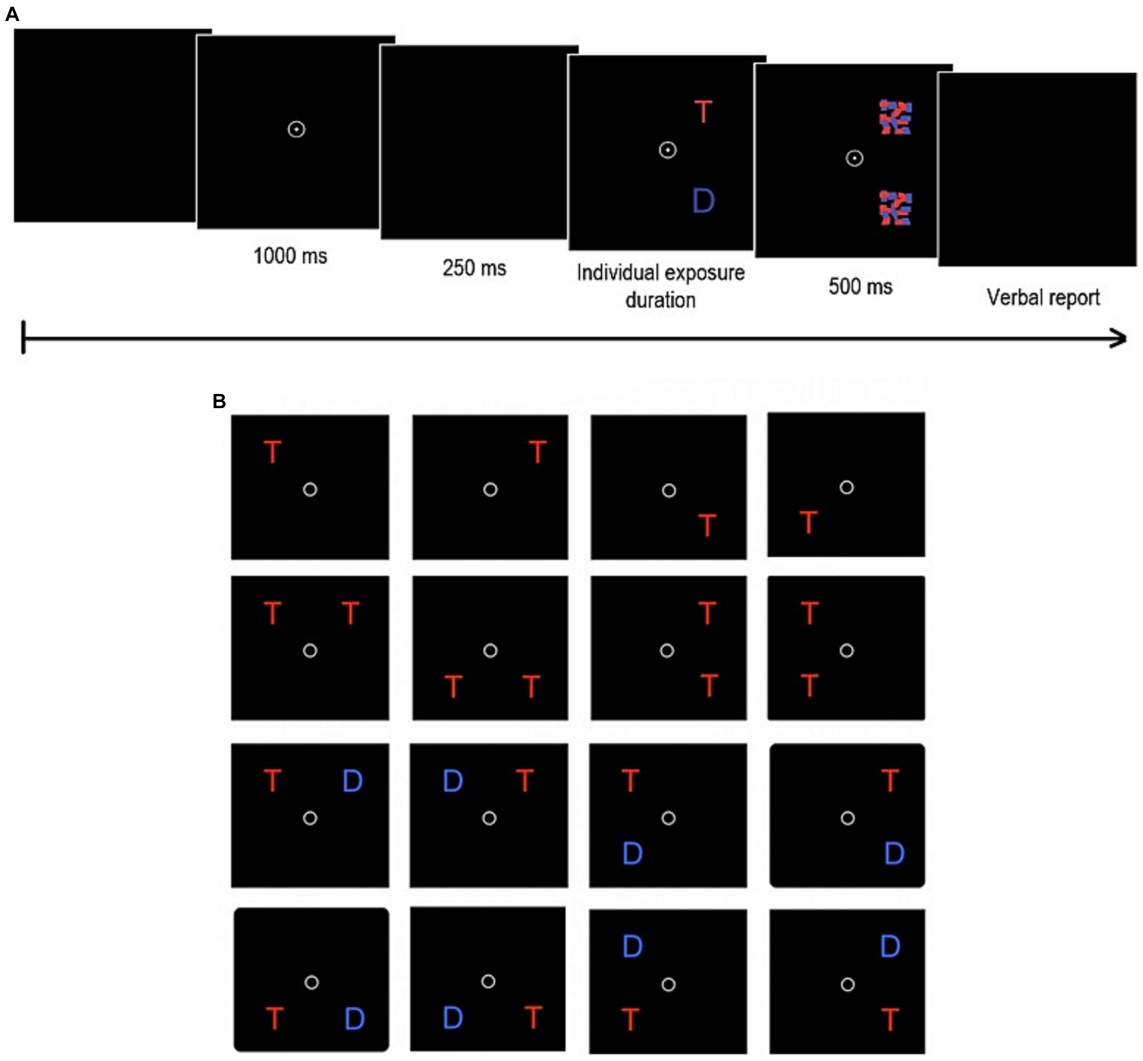
Trial sequence **(A)** and display types **(B)** of the TVA-based partial report task. After the presentation of a central fixation point for 1,000 ms and a brief delay of 250 ms, one of the 16 possible display types appears for a predetermined individual exposure duration. Following that, presented stimuli (*T* = target = red letters; *D* = distractor = blue letters) are masked for 500 ms before participants verbally report the letters.

In the partial report task, participants were instructed to only report target letters, which differed from distractors with respect to color (target = red; distractor = blue). In each trial, either a single target (letter) or a target plus distractor (letter) or two targets appeared at the corners of an imaginary square located 7.5 cm around the fixation point (see Figure 4A). All trials were followed by masks. If two letters were presented on the display, they were either flashed in a row or in a column, but never diagonally. In total, the partial report task consisted of 16 conditions, which were counterbalanced across all six blocks: target appearing alone (*T*; four possible alternatives: upper right/left or lower right/left corner), target appearing with distractor (*T*–*D*; eight possible alternatives) and two targets appearing together (*T*–*T*; four possible alternatives) (see Figure 4B). After establishing the participant’s individual exposure duration, the main task was started which consisted of 6 blocks with 48 trials each. Fitting of performance on the partial-report task delivers estimates of the attentional-selectivity parameter top-down control *α*. It indicates the relative attentional weights of distractors compared to targets (*w*D/*w*T). Targets receive more weight than distractors if *α* < 1. Accordingly, the lower the *α*-value, the more efficient the top-down control and the better the ability to prioritize targets over distractors.

### 2.4 Statistical analysis

We used standard descriptive statistics (e.g., means and percentages) to summarize the characteristics of the sepsis survivors and the age- and education-matched healthy controls. We performed a comprehensive check for outliers of continuous variables (outside of ±3 * interquartile range) and ran all analyses with and without outliers to check whether the results deviated. Next, we applied two-sample *t*-tests with equal variances to compare sepsis survivors and healthy controls regarding continuous demographic variables and neuropsychological and TVA-based outcomes. To estimate the magnitude of the observed deficits, we computed Cohen’s *d* (Cohen, 1988) for the differential parameter estimates between the two groups. Drawing on these results, we investigated whether the TVA-based parameters explained NAB outcomes. Afterwards we computed stepwise multiple linear regression analyses (without automated variable selection) using the NAB outcomes in which group differences were observed as the dependent variables in different models. In step 1, we included only the TVA-based parameters in which group differences were identified as predictors into each model. In step 2, we added group (sepsis survivors vs. healthy controls), age, sex, anxiety and depression as covariates in each model. Inclusion of age into these models is motivated by the observation that, while NAB scores are normed for age, processing speed (Habekost et al., 2013) and working memory storage capacity (McAvinue et al., 2012) decrease with age. We also included depression and anxiety as covariates due to the group differences in our samples (see Results) and consequently, to control for the influence of these differences on neuropsychological deficits. For all multiple regression analyses, we tested whether the assumptions were met. More specifically, we tested for multicollinearity of predictors (by inspecting VIFs) as well as independence (Durbin-Watson-test) and normality of residuals (Shapiro–Wilk-test). For all multiple regression analyses, the assumptions were met, as indicated by VIFs ranging from 1.063 to 1.727, Durbin Watson scores ranging from 1.674 to 2.113 and all *p*-values of Shapiro–Wilk-tests ≥0.131. Finally, we were interested in the relationship between TVA-based and NAB outcomes with medical data. Medical variables (i.e., ICU treatment days, ventilation days, days of delirium) were log-transformed as they were positively skewed. In order to better understand the relationship between TVA-based parameters and/or NAB outcomes with the medical variables, we also created scatter plots and computed Pearson correlations. As all analyses are exploratory, we did not correct for multiple testing and focus on estimates [point estimates and 95%-confidence intervals (CI)] as compared to “significance” roughly following the ongoing discussions (e.g., Amrhein et al., 2019). Statistical analyses were done in SPSS (IBM Corp. Released 2020. IBM SPSS Statistics for Windows, Version 27.0. Armonk, NY: IBM Corp).

## 3 Results

### 3.1 Demographic and medical data

Demographic and medical data of sepsis survivors and their age- and education-matched healthy controls are displayed in Table 1. While, we observed no evidence for group differences for visual contrast sensitivity or crystallized IQ, patients reported more depressive and anxiety symptoms compared to controls.

### 3.2 Clinical neuropsychological data: NAB

**Figure 5:**
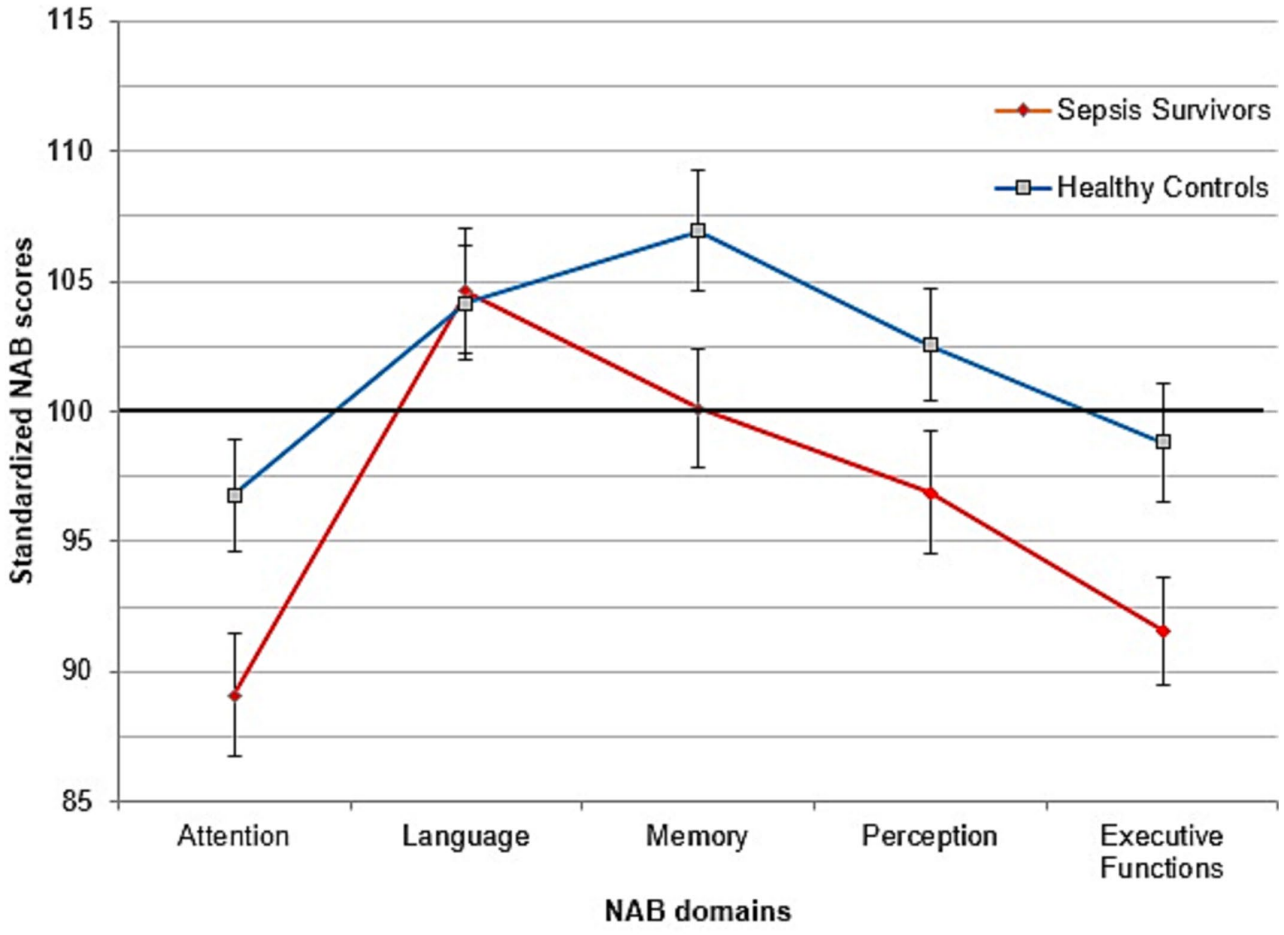
Profile from the Neuropsychological Assessment Battery for sepsis survivors (red) and controls (blue). Mean = 100, *SD* = 15. Error bars represent standard errors of the Mean.

Figure 5 presents the neuropsychological profile for sepsis survivors and healthy controls. Table 2 shows mean values and standard deviations for each single domain. Independent-sample *t*-tests revealed that sepsis survivors had lower Attention scores than healthy control participants (*t*(68) = 2.42, *p* = 0.018; 95%-CI [1.3–14.0]). The obtained value of Cohen’s *d* = 0.58, 95%-CI [0.10–1.05] indicated a moderate effect size of the difference in Attention between groups. The difference was most pronounced in the subtests “Digit span forwards” (i.e., repeating prolonged numerical series) and “Digits and letters” (i.e., quickly crossing out all letters ‘a’ in a large digit-letter array), both *p*-values ≤ 0.042. Sepsis survivors also showed lower Memory scores (*t*(68) = 2.10, *p* = 0.039; 95%-CI [0.3–13.3]). Cohen’s *d* = 0.50, 95%-CI [0.02–0.98] indicated a moderate effect size. This difference resulted from a lower subtest score in “immediate recall of a memorized story,” *t*(68) = 2.10, *p* = 0.039. Finally, sepsis survivors had lower Executive Functions scores than healthy control participants (*t*(68) = 2.32, *p* = 0.023; 95%-CI [1.0–13.4]). Cohen’s *d* = 0.56, 95%-CI [0.08–1.03] revealed a moderate effect size. Here, on the subtest level, patients scored lower than healthy control participants in the subtest “Mazes” (i.e., solving different mazes on paper), *t*(68) = 2.70, *p* = 0.009; 95%-CI [1.4–8.9]. No evidence for differences were observed for Language and Perception (both *p*-values ≥ 0.079).

**Table 2:** Results from the Neuropsychological Assessment Battery for sepsis survivors and healthy controls.

Cognitive domain	Sepsis survivors (*n* = 35)	Healthy controls (*n* = 35)	*T*-value	*p*-value*
Attention	89.1 (2.35)	96.8 (2.15)	2.42	0.018
Language	104.6 (2.39)	104.2 (2.18)	−0.14	0.888
Memory	100.1 (2.26)	106.9 (2.32)	2.10	0.039
Perception	96.9 (2.33)	102.5 (2.16)	1.78	0.079
Executive functions	91.6 (2.09)	98.8 (2.30)	2.32	0.023

Mean, SE in brackets. Standard scores (*M* = 100, SD = 15) for all variables.*Two-sided *p*-values from independent-sample *t*-tests.

### 3.3 Experimental neuropsychological data: TVA-based reports

#### 3.3.1 Whole-report results

Figure 3 presents the mean numbers of correctly reported letters as a function of the (effective) exposure duration in one representative participant from the sepsis survivors (male, 48 years) and one from the healthy control group (male, 44 years). The scores predicted by the TVA-model fits (approximately represented by the black dashed curves) and the observed scores were closely related. For each single participant in each group the accuracy of letter report as a function of exposure duration was modeled by a TVA function representing the maximum-likelihood fit to the data. This yielded individual estimates for perceptual processing speed *C*, perceptual threshold *t0* and working memory storage capacity *K* (group mean values and standard deviations are shown in Table 3). There was a close correspondence between the theoretically and the empirically obtained mean scores at the different exposure duration conditions (goodness-of-fit measures: sepsis survivors: *R*^2^ = 0.96; healthy controls: *R*^2^ = 0.85).

**Table 3:** Theory of Visual Attention-based Whole- and Partial-Report Parameters in sepsis survivors and healthy controls.

Parameter	Sepsis survivors (*n* = 35)	Healthy controls (*n* = 35)	*T*-value	*p*-value*
Visual processing speed *C*	27.47 (1.97)	28.29 (1.20)	0.36	0.723
Working memory storage capacity *K*	3.02 (0.11)	3.41 (0.11)	2.54	0.013
Perceptual threshold *t0*	26.26 (2.61)	20.13 (2.28)	−1.77	0.081
Top-down control *α*	0.57 (0.04)	0.47 (0.03)	−1.82	0.074

Mean, SE in brackets. C, elements per second; K, numbers of elements; t0, milliseconds.*Two-sided *p*-values from independent-sample *t*-tests.TVA, Theory of Visual Attention.

##### 3.3.1.1 Perceptual threshold

Figure 3 shows that, for both representative participants, the origin of the black dashed curve, *t0*, is located at similar values. TVA-model’s best fit to each participant’s data revealed average visual threshold *t0* estimates that are comparable between groups [*t*(67) = −1.77, *p* = 0.081].

##### 3.3.1.2 Visual processing speed

In Figure 3, also the initial slopes of the curves in *t0* of both representative participants are similar. Overall, we observed no evidence for group differences in visual processing speed *C* estimates between groups [*t*(67) = 0.36, *p* = 0.723] even after excluding one outlier in the patient group.

##### 3.3.1.3 Working memory storage capacity

**Figure 6:**
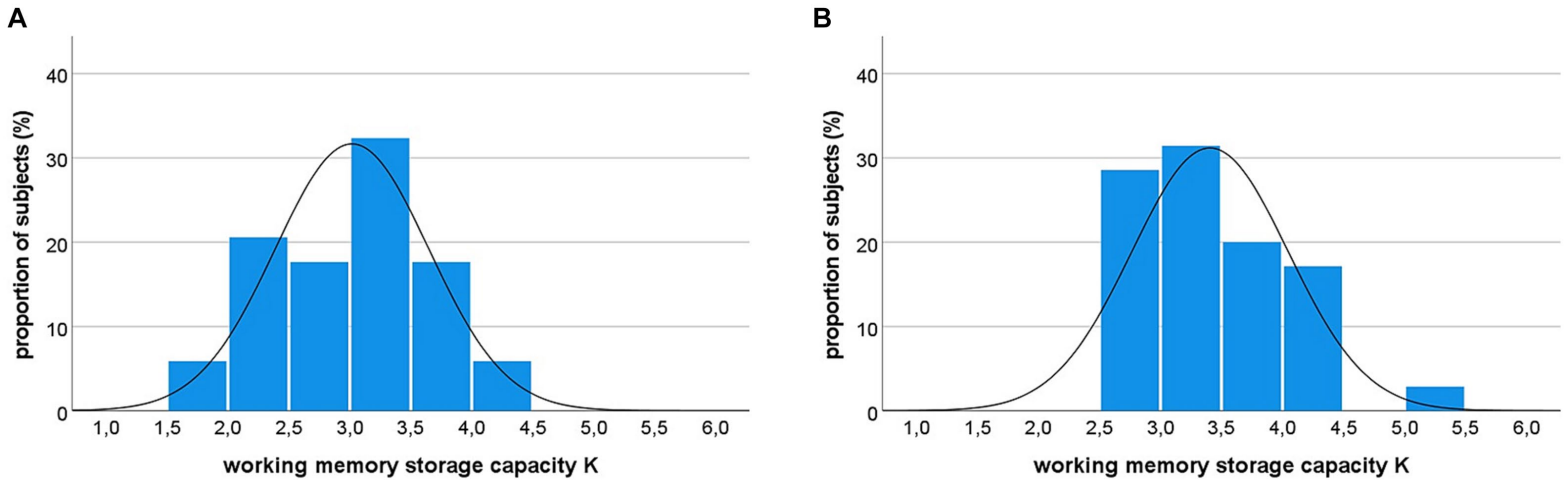
Distribution of working memory storage capacity *K* in sepsis survivors **(A)** and healthy controls **(B)**. Notably, 26% of sepsis survivors show a *K*-value below the range of healthy controls (i.e., *K* < 2.5).

Figure 3 shows that with prolonged exposure duration, an asymptotic level of reported letters is reached (indicated by the blue dashed horizontal line). This asymptote indicates the working memory storage capacity *K*, i.e., the maximum number of letters maintained in a given instance. It is higher for the representative healthy control participant compared to the sepsis survivor. Figure 6 presents the distribution of *K*-values for both groups. The sepsis survivors displayed lower working memory storage capacity *K* (*M* = 3.02, *SD* = 0.63) compared to heathy controls (*M* = 3.41, *SD* = 0.64) [*t*(68) = 2.54, *p* = 0.013]. The obtained value of Cohen’s *d* = 0.61, 95%-CI [0.13–1.09] indicated a moderate-to-large effect size of the difference in working memory storage capacity between groups.

#### 3.3.2 Partial report results

There was again a close correspondence between the theoretically and the empirically obtained mean scores at the different exposure duration conditions (goodness-of-fit measures: sepsis survivors: *R*^2^ = 0.62; healthy controls: *R*^2^ = 0.71).

##### 3.3.2.1 Top-down control

Average estimates of top-down control parameter *α* did not differ between groups [see Table 2; *t*(68) = −1.82, *p* = 0.074].

With respect to the question (i) raised in the introduction whether survivors of sepsis with cognitive impairments would suffer from changes in basic parameters we conclude from these results that sepsis survivors show reductions in working memory storage capacity. Other basic attentional parameters seem to be preserved.

### 3.4 Linear regression and correlative analyses

**Figure 7:**
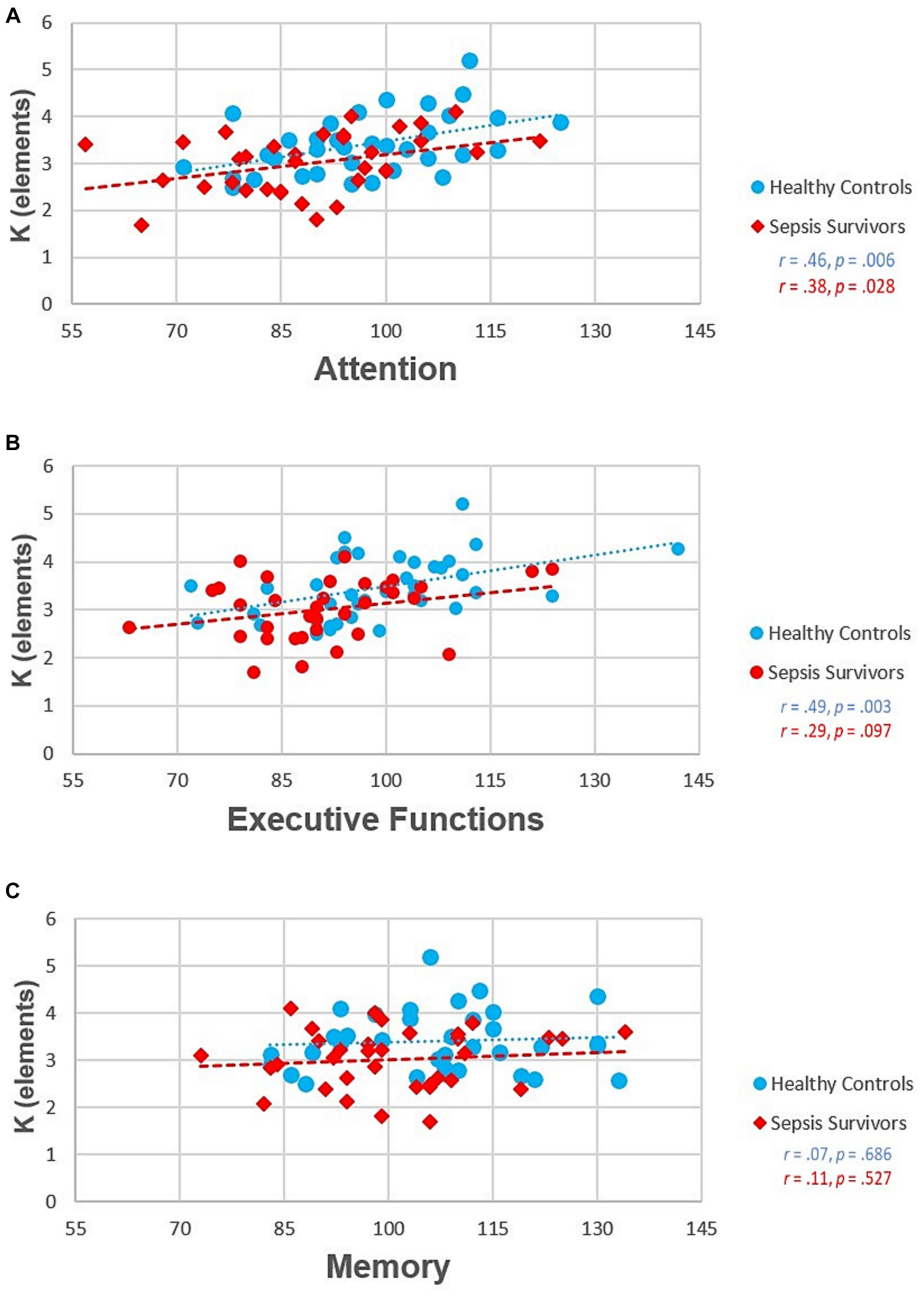
Associations between Neuropsychological Assessment Battery (NAB) domain subscores and working memory storage capacity *K*. **(A)** NAB Attention scores, **(B)** NAB Executive Function scores, **(C)** NAB Memory scores.

After identification of group differences in the working memory storage capacity *K*-parameter only, we were interested in its association with NAB outcomes. Thus, we first inspected relations between *K* and each of the NAB domains in which sepsis survivors performed worse (i.e., Attention, Memory, and Executive Functions) graphically via scatterplots (see Figure 7). We also ran three separate stepwise linear regression models (i.e., one for each NAB domain). In a first step, to identify the individual contribution of working memory storage capacity *K* on the NAB domains, we entered only *K* as predictor into each of the three models (Step 1). Subsequently, we added age, sex, group, depression and anxiety as predictors into each model to see if the results still held (Step 2).

#### 3.4.1 Attention

We observed an association between working memory storage capacity *K* with the NAB Attention score [*F*(1, 64) = 14.42, *p* ≤ 0.001; adjusted *R*^2^ = 0.174; see Table 4]. The extended/adjusted regression model in Step 2 yielded an adjusted *R*^2^ of 0.235 even though the 6% increase of explained variance could also be simply due to chance [change in *F*(5, 59) = 2.06, *p* = 0.083]. Furthermore, except for age (*p* = 0.011), none of the other predictors in the Step 2 model were associated with NAB Attention scores after mutual adjustment (all *p*-values > 0.342). However, further supplementary analyses showed that the effect of age is explained by the simultaneous inclusion of *K* in the regression model (data not shown).^4^ Hence, this finding may be explainable by the known, empirically demonstrated (McAvinue et al., 2012; Wiegand et al., 2014; Menegaux et al., 2020) negative correlation between age and *K* (*r* = −0.47, *p* < 0.001).

**Table 4:** Multiple stepwise linear regression on the Attention domain of the Neuropsychological Assessment Battery.

Variable	Regression coefficient B	SE	*β*	*T*-value	*p*-value
**Step 1: adjusted *R***^**2**^ **= 0.171**					
*K*	9.09 (4.31, 13.87)	2.39	0.43	3.80	<0.001
**Step 2: adjusted *R***^**2**^ **= 0.235, *p*(Δ*R***^**2**^**) = 0.083**					
*K*	11.91 (6.53, 17.30)	2.69	0.56	4.43	<0.001
Age	0.36 (0.08, 0.63)	0.14	0.33	2.62	0.011
Sex	−0.43 (−6.51, 5.65)	3.04	−0.02	−0.14	0.888
Group	−3.29 (−10.16, 3.58)	3.43	−0.12	−0.96	0.342
HADS-D depression	0.19 (−0.83, 1.21)	0.51	0.05	0.37	0.711
HADS-D anxiety	−0.31 (−1.51, 0.48)	0.46	−0.10	−0.66	0.509

Estimates, 95% Confidence Intervals in brackets.Sex: 0 = female; 1 = male. SE = Standard Error. β = Standardized Regression Coefficient. K = working memory storage capacity, numbers of elements; NAB, Neuropsychological Assessment Battery; HADS-D, Hospital Anxiety and Depression Scale – German version.

#### 3.4.2 Executive functions

Similarly, we observed an association of working memory storage capacity *K* with the NAB Executive Function score [*F*(1, 64) = 13.71, *p* ≤ 0.001; adjusted *R*^2^ = 0.164; see Table 5]. The observed 5% increase of explained variance in the Step 2 model (adjusted *R*^2^ = 0.217) was again compatible with chance [change in *F*(5, 59) = 1.87, *p* = 0.113]. Taking a closer look at the other Step 2 covariates none were associated with the Executive Functions score after mutual adjustment (all *p*-values > 0.088).

**Table 5:** Multiple stepwise linear regression on the Executive Functions domain of the Neuropsychological Assessment Battery.

Variable	Regression coefficient B	SE	*β*	*T*-value	*p*-value
**Step 1: adjusted *R***^**2**^ **= 0.164**					
*K*	8.90 (4.10, 13.71)	2.41	0.42	3.70	<0.001
**Step 2: adjusted *R***^**2**^ **= 0.217, *p*(Δ*R***^**2**^**) = 0.113**					
*K*	10.91 (5.46, 16.36)	2.72	0.51	4.00	<0.001
Age	0.20 (−0.07, 0.48)	0.14	0.19	1.48	0.114
Sex	0.98 (−5.17, 7.13)	3.07	0.04	0.32	0.750
Group	−1.88 (−8.83, 5.07)	3.47	−0.07	−0.54	0.590
HADS-depression	0.13 (−0.90, 1.15)	0.51	0.03	0.25	0.807
HADS-anxiety	−0.81 (−1.75, 0.13)	0.47	−0.25	−1.73	0.088

Estimates, 95% Confidence Intervals in brackets.Sex: 0 = female; 1 = male. SE = Standard Error. β = Standardized Regression Coefficient. K = working memory storage capacity, numbers of elements; NAB, Neuropsychological Assessment Battery; HADS-D, Hospital Anxiety and Depression Scale – German version.

#### 3.4.3 Memory

In contrast to the parameter related to Attention and Executive functions, *K* seemed to explain no variance in the NAB Memory Score [*F*(1, 64) = 1.65, *p* = 0.204; adjusted *R*^2^ = 0.010; see Table 6]. In contrast, the adjusted *R*^2^ for the Step 2 regression model was 0.159. Consequently, there was an increase of approximately 15% in explained variance [change in *F*(5, 59) = 3.27, *p* = 0.011]. After mutual adjustment, sex was the only covariate associated with NAB Memory Score (sex: *p* = 0.036; remaining predictors: *p*-values > 0.091).

**Table 6:** Multiple stepwise linear regression on the Memory domain of the Neuropsychological Assessment Battery.

Variable	Regression coefficient B	SE	*β*	*T*-value	*p*-value
**Step 1: adjusted *R***^**2**^ **= 0.010**					
*K*	3.44 (4.10, 13.71)	2.68	0.16	1.28	0.204
**Step 2: adjusted *R***^**2**^ **= 0.159, *p*(Δ*R***^**2**^**) = 0.011**					
*K*	3.32 (−2.35, 10.10)	2.89	0.15	1.15	0.256
Age	0.04 (−0.26, 0.33)	0.16	0.03	0.26	0.796
Sex	−7.00 (−13.52, − 0.47)	3.26	−0.25	−2.15	0.036
Group	−0.14 (−10.02, 6.03)	3.68	−0.01	−0.04	0.971
HADS-depression	−0.75 (−1.72, 0.64)	0.55	−0.20	−1.37	0.175
HADS-anxiety	−0.86 (−1.90, 0.25)	0.50	−0.26	−1.72	0.091

Estimates, 95% Confidence Intervals in brackets.Sex: 0 = female; 1 = male. SE = Standard Error. β = Standardized Regression Coefficient. K = working memory storage capacity, numbers of elements; NAB, Neuropsychological Assessment Battery; HADS-D, Hospital Anxiety and Depression Scale – German version.

Regarding questions (ii) and (iii) raised in the introduction, we found that parameter working memory storage capacity K predicts performance scores in the attention and executive functions domain of a standard neuropsychological battery and that it also accounts for the cognitive deficits in sepsis survivors compared to control participants.

#### 3.4.4 Medical data

There was no evidence for monotone correlations between medical variables and neuropsychological outcomes (all *r* < 0.32; all *p*-values ≥ 0.061; see Supplementary Table S1).

## 4 Discussion

Two key findings emerged from our study. Firstly, we demonstrated that working memory storage capacity, a crucial cognitive mechanism, is compromised in sepsis survivors exhibiting cognitive deficits. These patients are unable to maintain the same quantity of information at a given moment as their healthy counterparts. It is generally supposed that working memory capacity constitutes a major determinant of the maintenance of cognitive capabilities and functional independence in aging individuals (Salthouse, 1994; Cowan, 2010). The average estimate of visual working memory capacity of 3–4 elements in the healthy group is well in line with the typical estimates in other studies (Luck and Vogel, 1997; Cowan, 2001; Vogel and Machizawa, 2004; Finke et al., 2005). The substantial decline in working memory capacity, with a quarter of patients scoring outside the range of healthy controls, thus implies a substantial threat of independent functioning in our sample that can be regarded as representative of survivors of sepsis with cognitive deficits.

Secondly, our research indicates that this diminished capacity in working memory storage contributes significantly to the lower performance observed in standard neuropsychological tasks assessing attention and working memory. This is in line with a number of previous studies in healthy participants and patients with cognitive disturbances showing that the individual working memory capacity can account for a substantial amount of variance in performance in intellectual capabilities and various neuropsychological tasks (Cowan, 2010; Fukuda et al., 2010; Nyberg et al., 2012; Johnson et al., 2013; Luck and Vogel, 2013). Here, we demonstrated that the reduction in working memory storage capacity can explain why sepsis survivors underperform in attention tasks, such as digit span, and executive tasks, such as word fluency. While memory function impairments found in immediate recall in the sepsis survivors could also result from working memory reductions limiting the amount of information to be stored in a given instant, we did not find statistical support for a role of working memory in this score. Thus, in addition to working memory, additionally longterm memory, driven by other neural mechanisms, might lead to the reduced memory domain score.

Importantly, for attention and executive functions, the regression model indicated that *K* was an independent predictor even after adjusting for group affiliation. Thus, the performance differences between survivors of sepsis and healthy control participants may be fully explainable on the basis of the difference in *K*. Furthermore, it was found that other potential contributors to cognitive performance in sepsis survivors, such as age, sex, depression, and anxiety, were not of critical relevance, while working memory capacity alone stuck out as independent predictor of cognitive impairment. Importantly, e.g., the psychological burden, which is typically enhanced in survivors of sepsis, as documented also in the present study, was not found to contribute to the impaired neuropsychological task performance. Moreover, supplementary analyses showed that the effect of age on the attention domain is explained by the simultaneous inclusion with *K* into the regression model. Crucially, this effect is absent when entering age as a single predictor into the model which further strengthens the predictive power of *K.*

While we cannot establish a direct causal link between the observed cognitive deficits and sepsis in our sample, identifying the impairment in working memory and its impact on neuro-cognitive task performance is a critical advancement. It offers a testable, quantitative measure of a potentially underlying mechanism of sepsis-related impairment that can also be used in further studies in order to gain a better mechanistic understanding of the cognitive problems in survivors of sepsis. This insight is of clinical importance, as it directs attention to working memory as a specific target for potential therapeutic interventions, particularly for patients with confirmed working memory deficits. There is mounting evidence that working memory training is effective in enhancing not only working memory functions themselves (Jaeggi et al., 2008; Klingberg, 2010), but that it could also stimulate cognitive functioning on a more general level in younger (Au et al., 2015) and older persons (Karbach and Verhaeghen, 2014; Zinke et al., 2014).

Once identified as a central contributor to the cognitive problems found in survivors of sepsis with cognitive deficits, furthermore, the controlled, parameterized assessment of working memory provides a quantitative cognitive biomarker which offers the opportunity to more systematically assess the cognitive consequences of, e.g., diverse treatment options in sepsis patients applied also in the acute stage.

However, one needs to mention that the present study has several limitations. First, due to its cross-sectional study design it was possible to exclude patients with dementia diagnosis prior to sepsis, but not to estimate the degree of potential mild cognitive impairment prior to sepsis. Second, as we did not include a control group of non-sepsis post-ICU patients, it is unclear if the observed cognitive impairments result from the sepsis or from the ICU treatment. Thus, the observation of the relationship between working memory and standard cognitive performance might not be specific for sepsis survivors, but more generally found in ICU survivors. Third, because we included only patients with cognitive deficits according to the neuropsychological standard test results, our results might not be generalizable to all survivors of sepsis. Fourth, we are not able to estimate to which extent study participants can be thought of as representative for the overall population because we are not authorized to report on unpublished sociodemographic and clinical data from the whole MSC. It is not possible to cancel out the possibility that the overall more mobile and fitter people, both physically and mentally, have answered the call for study participation. Hence, there might be a recruitment bias. Fifth, sample sizes were quite small in both groups. Sixth, although it is known that cognitive dysfunctions leads to functional loss in family life and a reduced capability to return to work we cannot directly conclude from our results that the reduction in working memory capacity does lead to such functional loss. Last, our study does not provide neuroimaging data which could provide a neural basis for further elucidating the characteristics of working memory impairments in sepsis survivors.

### 4.1 Conclusion

In summary, we found that cognitively compromised survivors of sepsis showed persistent deficits in working memory storage capacity when measured with a comprehensive parameterized assessment based on the TVA. This reduction in working memory was strongly associated with outcomes of attentional and executive functions in a standard neuropsychological battery, even when controlling for demographic and clinical variables. These results suggest that working memory might be a critical determinant in the neurocognitive sequelae of sepsis.

## Data availability statement

The raw data supporting the conclusions of this article will be made available by the authors, without undue reservation.

## Ethics statement

The studies involving humans were approved by the Ethics Committee of the University Hospital Jena (Reg.-Nr. 2019-1411_1-BO). The studies were conducted in accordance with the local legislation and institutional requirements. The participants provided their written informed consent to participate in this study. They received monetary compensation for their participation.

## Author contributions

FK: Formal analysis, Investigation, Methodology, Writing – original draft. EH: Conceptualization, Investigation, Methodology, Writing – review & editing. CG: Conceptualization, Funding acquisition, Writing – review & editing. AS: Conceptualization, Formal analysis, Writing – review & editing. JW: Conceptualization, Investigation, Writing – review & editing. KF: Conceptualization, Funding acquisition, Methodology, Resources, Supervision, Writing – original draft.

## Footnotes

^1^STARDUsT is a randomized controlled pilot trial with a focus on an internet-based cognitive training intervention—the findings related to the training outcomes are not reported here.

^2^During the face-to-face assessment, this patient indicated an incident of stroke in his medical history.

^3^In a study on validity and norm equivalency of MWT-B the authors found MTWB scores to overestimate verbal IQ of the HAWIE-R (Revised German version of the Wechsler Intelligence Scale) by 17 points.

^4^As suggested by one reviewer and in order to doublecheck our assumption, we re-ran the regression analyses by entering only age in the first step and adding all other covariates including *K* in the second step. As expected, age *per se* did not predict any variance (all *r* < 0.05; all *p*-values ≥0.712).
