# Supplementary material for: Persistent neurocognitive deficits in cognitively impaired survivors of sepsis are explained by reductions in working memory capacity
Source: Front Psychol. 2024 Feb 21;15:1321145. doi: 10.3389/fpsyg.2024.1321145 (PMC10915060; doi:10.3389/fpsyg.2024.1321145)
Supplement: Supplementary file 1 [file Data_Sheet_1.pdf]

Supplementary Material

1. Supplementary Methods

1.1 Whole- and Partial Report General Procedure

General information. Both whole-report and partial-report paradigms were conducted in a dimly lit cabin. All stimuli were presented on an Asus 24-inch monitor (1980 × 1080 pixel screen resolution, 100 Hz refresh rate). Viewing distance was approximately 50 cm, controlled by the aid of a chinrest. The total number of trials was 288 in the partial-report, separated into blocks of 48 trials, and 336 in the whole-report experiment, presented in 4 blocks of 84 trials assigned randomly to a cue and no-cue condition (see below). Within each block, the different trial types were presented equally often in randomized order.

Masking. The masks consisted of squares matching the size of the letters (max.1.8 × 1.8 cm) filled with red and blue colored blobs. They were presented for 500 ms.

1.2 Whole- and Partial-Report Accuracy Rating

In order to avoid too conservative performance and guessing behavior, at the end of each test block, the experimenter was presented with an accuracy rating on a scale based on all reported letters. In case the accuracy level of the reported letters was outside a range of 70% to 90%, slightly adapted instructions were given for the next block. In case the participant's accuracy rating exceeded 90%, they were asked to also name letters that they believed to have recognized without being completely certain about it. If the accuracy score was lower than 70%, the participants were instructed to report letters more conservatively, i.e. to report only those letters they have recognized with a high certainty even if that led to reporting less letters overall.

1.3 Whole-Report Pretest Procedure

Throughout all trials in the whole-report procedure, the color of the letters varied randomly between red and blue with all 6 letters always appearing in the same color per trial. The pretest phase consisted of 48 trials in total, divided into 4 practice blocks with an accuracy rating following each block. The 12 trials in each block were composed of 4 triplets of trials. Each triple contained one masked calibration trial and 2 trials with long exposure durations, either masked with 250 ms or unmasked with 200 ms exposure duration, to not discourage the participant. Trials within each triple were randomized. The calibration trials started with an exposure duration of 100 ms. If the participant was able to report at least one of the 6 letters correctly, the exposure duration was decreased by 10 ms. In case none of the letters was named correctly, the exposure duration was increased by 10 ms. At the end of the adjustment block, the lowest adjusted exposure duration for the following test phase was determined as the shortest exposure duration used in the calibration trials. Hence, participants were not able to report any letter with this exposure duration. Four additional exposure durations were picked from a predefined list based on that value (e.g., if lowest exposure duration was 20 ms, adjusted exposure duration (AEDs) = [20, 40, 60, 120, 210]; if it was 90 ms, AEDs = [90, 120, 160, 210, 280]). In addition to masked versions of these exposure durations from the pretest, two exposure were also presented without masks. Hence, this resulted in seven effective exposure durations, producing a broad spectrum of performance by covering near-threshold and maximum performance levels.

1.4 Whole-Report Cueing Procedure

In half of the trials, an auditory cue (an 80 dB tone) was administered 200 ms prior to stimulus presentation. This “alerting” cue was not of relevance to the current analysis, but served for the evaluation of a training procedure applied to patients that intended

to enhance alertness, among other attentional functions. Cue- and no-cue conditions were therefore not analyzed separately in the current study (see 2.1).

1.5 Partial Report Pretest Procedure

First, 24 trials with an initial exposure duration of 80 ms each were presented. This was decreased by steps of 10 ms if participants were able to report two letters in the target–target condition. In contrast, if they could only name one of the two target letters, exposure duration was kept at 80 ms, and if none of the two targets could be reported, exposure duration was increased by steps of 10 ms until participants could name, on average, one letter per trial correctly. Subsequently, 24 trials were run and performance at the calculated exposure duration was checked for the different experimental conditions. If participants reported 70–90% of the single targets (*T*) and at least 50% of the dual targets (*T*–*T*) correctly, the selected exposure duration was kept for the main partial report task. If not, exposure durations were increased (if performance was below those percentages) or decreased (if performance was above 90% in the T condition) manually by the experimenter and performance was rechecked in another round of 24 trials. The patient group's average exposure duration determined in the pre-test (for the detailed procedure see Supplementary Material) was 108.00 ms (SD = 43.51), and it did not differ ($t(68) = -1.50$, $p = .14$) from that of the control group (mean = 94.86 ms, SD = 28.01).

1 Supplementary Results

2.1 Cueing Effects in TVA-based analyses

A 2 × 2 mixed ANOVA with the within-factor Cue (Cue *t0*, -*C*, -*K* vs. No Cue *t0*, -*C*, -*K*) and the between-factor Group (sepsis survivors vs. healthy controls) yielded no significant interaction, $F(3, 65) = .42$, $p = .740$. There was, however, a significant main effect for Cue *C*, $F(3, 65) = 24.78$, $p < .001$, partial $\eta^2 = .53$. In more detail, cueing led to a significant increase in visual processing parameter C, $F(1, 67) = 18.05$, $p < .001$, partial $\eta^2 = .21$. There were no main effects for *t0* and *K* (both *p*-values > .149). Consequently, as we were interested in the overall effect for main analyses, we computed the average of the respective cue- and no-cue parameters for each participant (e.g. mean C = Cue C + No-Cue C /2). All consecutive analyses were run with these averages.

Table S1

Bivariate Pearson correlations between Neuropsychological Assessment Battery cognitive domain subscores, Theory of Visual Attention parameters & medical data in sepsis survivors.

Variable	Days of delirium	ICU treatment days	Ventilation days	Time interval since ICU discharge in months
Attention	.16 (.36)	-.16 (.37)	-.21 (.22)	.23 (.19)
Memory	-.02 (.93)	-.15 (.40)	-.09 (.61)	.10 (.58)
Executive Functions	.22 (.20)	-.03 (.86)	-.08 (.67)	.29 (.10)
Visual Processing Speed *C*	-.07 (.71)	-.13 (.48)	-.06 (.74)	.25 (.16)
Working Memory Storage Capacity *K*	.10 (.59)	-.24 (.18)	-.18 (.32)	-.08 (.67)
Perceptual Threshold *t0*	.16 (.38)	-.14 (.44)	-.23 (.20)	-.12 (.49)
Top-Down-Control α	-.21 (.21)	-.25 (.14)	-.24 (.18)	.32 (.06)

Note*:* *p*-values in parentheses. ICU = Intensive Care Unit

Table S2

Demographic and medical data in original MSC sample and STARDUsT sample.

Characteristic	Original MSC sample (n = 907)	STARDUsT sample (*n* = 35)
Age (years)	65 (56-74)	58 (48-70)
Male sex	584 (64.4%)	21 (60.0%)
ICU treatment (days)	10 (4-26)	11 (5-26)
Duration of Ventilation (days)	6 (2-16)	6 (2-16)
Duration of delirium (days)	4 (2-9)	2 (2-7)

Note. Absolute and relative frequencies or median with first and third quartile are provided. MSC data taken from Fleischmann et al. (2021)
